# Patient perspectives on simultaneous methotrexate–folic acid intake to improve adherence in rheumatoid arthritis: a cross-sectional survey

**DOI:** 10.1093/rap/rkag109

**Published:** 2026-10-08

**Authors:** Elisa Dalix, Naïma Hamamouche, Irène Pico Philippe, Sandrine Rollot, Hubert Marotte

**Affiliations:** SAINBIOSE U1059, University of Jean Monnet Saint-Etienne, Mines Saint-Etienne, INSERM, Saint-Etienne, France; Sanoïa, e-Health Services, Aubagne, France; AFPric (Association Française des Polyarthritiques et des Rhumatismes Inflammatoires Chroniques), Paris, France; AFPric (Association Française des Polyarthritiques et des Rhumatismes Inflammatoires Chroniques), Paris, France; Department of Rheumatology, SAINBIOSE U1059, University of Jean Monnet Saint-Etienne, CHU Saint-Etienne, Mines Saint-Etienne, INSERM, Saint-Etienne, France

**Keywords:** rheumatoid arthritis, methotrexate, folic acid, medication adherence, surveys and questionnaires

## Abstract

**Objective:**

To assess real-world adherence to folic acid (FA) supplementation with methotrexate (MTX) and patient perceptions of a simplified, same-day dosing strategy in RA.

**Methods:**

We conducted a cross-sectional online survey co-designed with patient representatives and distributed by the French patient association AFPric. RA patients treated with MTX were eligible. The questionnaire captured MTX and FA regimens, omissions, adverse events and attitudes toward same-day MTX and FA administration. Analyses were descriptive with prespecified subgroup comparisons.

**Results:**

Among 967 respondents (86.8% women; mean age 62.4 years), 86.7% reported once-weekly FA supplementation. Overall, 25.5% reported at least occasional FA omission, with omissions more frequent among long-term MTX users. Nausea after MTX was reported by 46.6% of respondents and by 52.6% of those reporting FA omissions. A same-day MTX and FA regimen was perceived as simpler by 30.5% and 41.5% believed it could reduce FA omission. Overall, 64.8% indicated that they would be willing to participate in a randomised controlled trial evaluating simultaneous administration.

**Conclusion:**

One in four people with RA treated with MTX reported FA omissions. Many respondents believed that a same-day regimen could reduce FA omissions, and interest in participating in a clinical trial was high. These findings support the feasibility of prospectively evaluating whether same-day MTX and FA administration may improve adherence and tolerability in selected patients, particularly those receiving once-weekly FA supplementation, without compromising MTX effectiveness.

Key messages

- Folic acid omissions were reported by one in four methotrexate-treated patients.
- Many patients believed same-day methotrexate–folic acid administration could reduce folic acid omissions.
- A randomised trial is warranted to evaluate same-day methotrexate–folic acid administration.

## Introduction

Methotrexate (MTX) is the anchor conventional synthetic DMARD (csDMARD) for RA, owing to its proven efficacy, safety and cost-effectiveness [1–3]. It is recommended by major rheumatology societies, including the EULAR [2] and the French Society of Rheumatology [3], and it is listed as an essential medicine by the World Health Organization [1]. MTX is typically administered once weekly and remains the only DMARD associated with reduced all-cause mortality in RA [4, 5].

Folic acid (FA) supplementation is routinely recommended to reduce MTX-related toxicity. However, both dosing and timing vary widely across clinical practice. In routine clinical practice, FA is commonly prescribed orally on one or more days following MTX administration, reflecting the absence of harmonised guidance on optimal scheduling. Beyond its role in reducing toxicity, FA may also interact with MTX pharmacodynamics, including the potential effect on gut microbiota homeostasis [6]. Importantly, preclinical data suggest that simultaneous MTX and FA administration does not impair MTX efficacy compared with delayed FA intake [7].

Despite its strong efficacy, 7–30% of patients discontinue MTX within the first year of treatment, most commonly due to adverse events, particularly gastrointestinal, hepatic and haematological effects [8–10]. FA deficiency is implicated in several of these adverse effects. Even at low doses, FA supplementation reduces MTX-related gastrointestinal and liver toxicity [11] and is associated with lower MTX discontinuation rates [12]. Adherence to FA supplementation is therefore clinically important, as it may facilitate long-term MTX persistence and improve treatment tolerability.

However, adherence to FA supplementation in routine practice remains poorly characterised and little is known about patient perceptions of alternative, potentially simpler dosing strategies. From an implementation perspective, simplifying FA administration, such as through same-day MTX and FA intake, could reduce omissions and improve adherence, but patient acceptability of such strategies has not been systematically explored.

To inform the design of a future clinical trial evaluating simultaneous MTX and FA administration, we conducted a cross-sectional, patient co-designed survey among individuals with RA in France. Our objectives were to describe real-world MTX and FA intake patterns, identify adherence challenges, and assess patient attitudes toward same-day MTX–FA administration.

## Methods

### Survey design

We conducted a cross-sectional, patient-co-designed online survey on the SANOÏA platform, in collaboration with the French patient association AFP^ric^ (Association Française des Polyarthritiques et des Rhumatismes Inflammatoires Chroniques). The questionnaire was co-developed by patient representatives, researchers and rheumatologists. The survey was disseminated on 25 October 2023 and remained open for 12 days.

### Participants

Eligible respondents were adults with RA currently treated with MTX. Exclusion criteria included age <18 years, missing consent, unconfirmed RA diagnosis or an absence of FA supplementation. Of 1238 responses received, 967 were included in the final analysis after applying prespecified exclusions.

### Questionnaire and variables

The survey collected data on demographics, disease characteristics, MTX exposure and regimen, FA supplementation patterns, adherence behaviours, MTX-related adverse effects (including nausea within 48 h of MTX intake) and patient preferences regarding same-day MTX–FA administration, including willingness to participate in a randomised controlled clinical trial. The frequency of FA omission was assessed using a 5-point Likert-type scale commonly used in self-administered questionnaires. Participants reported the frequency of omission as ‘all the time’, ‘regularly’, ‘sometimes’, ‘occasionally’ or ‘never or rarely’. These response options were presented verbatim to participants and were not anchored to predefined numerical frequencies.

### Statistical analysis

Analyses were performed using R software (version R 4.3.1, R Foundation for Statistical Computing, Vienna, Austria). Descriptive statistics were used to summarise the data. Group comparisons were conducted using Kruskal–Wallis tests for continuous variables and Fisher’s exact or Pearson’s chi-squared tests for categorical variables, as appropriate. Two-sided *P*-values <0.05 were considered statistically significant. Prespecified subgroup analyses were performed according to MTX treatment duration (0–2, 2–10, >10 years).

### Ethical considerations

The study complied with French regulations governing non-interventional research. Participants were informed about the study and data-protection measures. No identifying data were collected and all data were stored on an ISO 27001–certified secure health data platform. On this basis, institutional review board or ethics committee approval was not required.

## Results

### Demographics and treatment characteristics

The study population reflected demographic characteristics typical of patients with RA in France (Table 1), with a mean age of 62.4 years (s.d. 11.3) and a predominance of women (86.8% of respondents). Mean age increased with a longer duration of MTX treatment (*P* < 0.001) while sex distribution did not differ across MTX exposure categories. MTX treatment duration was heterogeneous. Nearly half of participants (49.4%) had been treated with MTX for >10 years. For analysis, MTX exposure was grouped into 0–2 years (12.4%), 2–10 years (38.1%) and >10 years (49.4%). Nausea within 48 h of MTX administration was reported by 46.6% of respondents. The occurrence of nausea did not differ significantly according to MTX treatment duration (NS).

**Table 1:** Patient characteristics according to MTX duration and FA supplementation.

Characteristics	MTX duration				
	Overall (*N* = 967)	0–2 years (*n* = 120)	2–10 years (*n* = 369)	>10 years (*n* = 478)	*P*-value
Age, years, mean (s.d.)	62.4 (11.3)	56.4 (11.4)	60.2 (11.4)	65.6 (10.2)	<0.001^a^
Gender, *n* (%)					
Male	128 (13.2)	11 (9.2)	49 (13.3)	68 (14.2)	
Female	839 (86.8)	109 (90.8)	320 (86.7)	410 (85.8)	0.4^b^
MTX duration, *n* (%)					
<6 months	28 (2.9)	28 (23.3)			
6–12 months	33 (3.4)	33 (27.5)			
1–2 years	59 (6.1)	59 (49.2)			
2–5 years	185 (19.1)		185 (50.1)		
5–10 years	184 (19.0)		184 (49.9)		
>10 years	478 (49.4)			478 (100)	
Nausea within 48 h after MTX, *n* (%)					
Yes	451 (46.6)	57 (47.5)	185 (50.1)	209 (43.7)	
No	516 (53.4)	63 (52.5)	184 (49.9)	269 (56.3)	0.2^c^
Weekly FA, *n* (%)					
<1	12 (1.2)	1 (0.8)	4 (1.1)	7 (1.5)	
1	838 (86.7)	107 (89.2)	329 (89.2)	402 (84.1)	
2	46 (4.8)	8 (6.7)	18 (4.9)	20 (4.2)	
3	18 (1.9)	1 (0.8)	4 (1.1)	13 (2.7)	
4	5 (0.5)	0	0	5 (1.0)	
5	26 (2.7)	2 (1.7)	7 (1.9)	17 (3.6)	
6	22 (2.3)	1 (0.8)	7 (1.9)	14 (2.9)	0.2^c^
FA omission, *n* (%)					
Yes	247 (25.5)	19 (15.8)	89 (24.1)	139 (29.1)	
No	720 (74.5)	101 (84.2)	280 (75.9)	339 (70.9)	<0.01^c^

The percentages are expressed based on the number of patients per group of MTX treatment duration.

a Kruskal–Wallis rank sum test.

b Fisher’s exact test.

c Pearson’s chi-squared test.

### FA supplementation and adherence

Most patients (86.7%) reported once-weekly FA supplementation. A non-significant trend toward a greater number of days of FA intake was observed in patients treated with MTX for >10 years. Overall, 25.5% of respondents omitted FA at least occasionally. FA omissions increased with MTX treatment duration (0–2 years: 15.8%; 2–10 years: 24.1%; >10 years: 29.1%; *P* < 0.01).

Among the 247 respondents reporting FA omission, 38.5% selected ‘never or rarely’, 35.2% ‘occasionally’, 16.2% ‘sometimes’, 9.3% ‘regularly’ and 0.8% ‘all the time’ (Supplementary Table S1).

Among respondents reporting FA omission, 52.6% experienced nausea within 48 h after MTX intake. Nausea was more frequently reported among those with shorter MTX exposure (0–2 years: 68.4%) compared with those treated for 2–10 years [55.1% (NS)] or >10 years [48.9% (NS)]. An increase in nausea after FA omission was reported by 16.2% of respondents and tended to be more frequent in patients initiating MTX (0–2 years: 31.6%; 2–10 years: 19.1%; >10 years: 12.2%; *P* = 0.06).

### Patient preferences regarding MTX and FA administration

Perceptions of same-day MTX and FA administration are shown in Table 2. Overall, 30.5% of respondents perceived same-day intake as simpler, a perception that was numerically more frequent among patients with shorter MTX exposure (*P* = 0.1). Regarding prevention of FA omissions, 41.5% of respondents agreed or strongly agreed that same-day intake could improve adherence, 43.4% were indifferent and 15.1% disagreed or strongly disagreed. Responses did not differ according to MTX treatment duration (*P* = 0.2). There was a high level of willingness to participate in a clinical study evaluating the same-day administration of MTX and FA (64.8%), with similar proportions across MTX exposure categories (*P* = 0.4).

**Table 2:** Preferences regarding same-day MTX–FA dosing and readiness for a randomised trial.

Preferences	MTX duration				
	Overall (*N* = 967)	0–2 years (*n* = 120)	2–10 years (*n* = 369)	>10 years (*n* = 478)	*P*-value
Taking FA on the same day as MTX would be, *n* (%)					
Much simpler/simpler	295 (30.5)	42 (35)	122 (33.1)	131 (27.4)	
No change/less simple	672 (69.5)	78 (65)	247 (66.9)	347 (72.6)	0.1
Taking FA on the same day as MTX would prevent FA omission, *n* (%)					
Strongly agree/agree	401 (41.5)	57 (47.5)	148 (40.1)	196 (41.0)	
Indifferent	420 (43.4)	44 (36.7)	174 (47.2)	202 (42.3)	
Disagree/strongly disagree	146 (15.1)	19 (15.8)	47 (12.7)	80 (16.7)	0.2
Willingness to participate in a clinical study to evaluate the combination of MTX and FA, *n* (%)					
Yes	627 (64.8)	78 (65)	250 (67.8)	299 (62.6)	
No	340 (35.2)	42 (35)	119 (32.2)	179 (37.4)	0.4

*P*-values were calculated using Pearson’s chi-squared test.

## Discussion

Our study provides patient-centred real-world data on FA supplementation among patients with RA receiving MTX. Three main findings emerge from this survey. First, approximately one-quarter of respondents reported at least occasional FA omission, despite the established role of FA supplementation in reducing MTX-related toxicity [11, 12]. Second, self-reported FA omission was more frequent with an increasing duration of MTX treatment. Third, although same-day MTX and FA administration was not perceived as simpler by most respondents, a substantial proportion believed that it could reduce FA omissions, and nearly two-thirds expressed a willingness to participate in a randomised trial evaluating this strategy.

FA supplementation is generally considered a simple adjunct to MTX therapy, yet our findings suggest that adherence to supportive medication should not be assumed. Overall, 25.5% of respondents reported at least occasional FA omission. Although most reported omissions were infrequent, this finding may be clinically relevant because folate supplementation reduces MTX-related gastrointestinal and hepatic toxicity and may reduce MTX discontinuation [11, 12]. Importantly, our survey relied on self-reported medication-taking behaviour rather than a validated adherence instrument or objective adherence measure. The observed proportion should therefore be interpreted as a patient-reported estimate of FA omission rather than as a precise measure of medication adherence.

Interestingly, FA omission was more frequently reported with an increasing duration of MTX treatment, from 15.8% among patients treated for 0–2 years to 29.1% among those treated for >10 years. This association may suggest that maintaining adherence to supportive medication becomes more difficult during long-term treatment. Treatment burden, established routines or reduced attention to an adjunctive medication could potentially contribute to this pattern. However, these mechanisms were not assessed in our questionnaire, and the cross-sectional design precludes attributing the observed association to treatment duration itself. In particular, disease activity was not collected, and patients with well-controlled disease may perceive some components of their treatment as less necessary. Longitudinal studies would be required to determine whether FA adherence actually changes over the course of MTX treatment.

The optimal timing of FA supplementation relative to MTX administration remains uncertain and recommendations are not uniform. French recommendations support systematic FA supplementation with MTX but do not specify a particular interval between FA and MTX administration [3]. Similarly, current European and American recommendations do not provide a specific evidence-based schedule for separating FA from the weekly MTX dose [2, 13]. In contrast, the British Society for Rheumatology recommends that FA should not be taken on the day of MTX administration, while acknowledging the limited evidence regarding the optimal timing of supplementation [14]. Similarly, Japanese recommendations advise administering FA 24–48 h after MTX [15]. Thus, although temporal separation of MTX and FA is common in clinical practice, its superiority over same-day administration has not been established. In addition, our previous preclinical study found no impairment of MTX efficacy when MTX and FA were administered simultaneously compared with separate administration [7]. Together, these uncertainties provide a rationale for prospectively evaluating whether same-day administration could simplify treatment without compromising MTX effectiveness.

The patient perspective on same-day MTX and FA administration provides an additional insight. Our survey did not evaluate whether patients actually taking MTX and FA on the same day had better adherence. Rather, it assessed how patients perceived such a strategy. Only 30.5% considered same-day administration simpler, while 41.5% believed that it could reduce FA omissions. These findings should therefore not be interpreted as evidence of a general patient preference for same-day administration or of its effectiveness in improving adherence. Patient-perceived simplicity and actual medication-taking behaviour are distinct outcomes and should both be evaluated prospectively. Nevertheless, 64.8% of respondents indicated that they would be willing to participate in a randomised clinical trial evaluating simultaneous administration. This high level of willingness suggests that the question is relevant to patients and supports the feasibility of prospectively testing this strategy.

However, same-day administration would not be appropriate for all patients. Although 86.7% of respondents reported once-weekly FA supplementation, a minority received FA on several days per week. For these patients, consolidating FA supplementation on the day of MTX administration may be impractical or clinically inappropriate. Our questionnaire recorded the number of weekly FA administrations but not the dose administered at each intake, the total weekly dose or the clinical rationale underlying the prescribed regimen. We therefore cannot determine whether more frequent supplementation reflected MTX-related adverse events, comorbidities, physician preference or other patient-specific considerations. Any future trial of same-day administration should consequently define eligible patients according to their usual FA regimen rather than consider this strategy as universally applicable.

Nausea within 48 h of MTX administration was reported by 46.6% of the overall population and by 52.6% of respondents reporting FA omission. Among patients reporting omissions, 16.2% perceived increased nausea when FA was omitted. This observation is biologically and clinically plausible given the established protective effect of folate supplementation against MTX-related gastrointestinal toxicity [11, 12], but should be interpreted cautiously. The questionnaire did not collect information on symptom duration or severity, concomitant medications, comorbidities, disease activity or the route of MTX administration. Furthermore, the cross-sectional design does not allow the temporal or causal relationship between FA omission and nausea to be established. FA omission may contribute to poorer tolerability, but conversely, patients experiencing treatment-related symptoms may have different medication-taking behaviours. Therefore, residual confounding and recall bias cannot be excluded.

Several other limitations should be acknowledged. First, all data were self-reported, making them susceptible to recall and social desirability biases. Second, recruitment through a national patient association may have selected patients who are particularly engaged in their disease management and may limit generalizability to other RA populations or healthcare systems. Third, specific reasons for FA omission were not collected, preventing us from distinguishing unintentional omission from intentional non-adherence or from identifying treatment-related determinants of missed doses. Fourth, we did not collect disease activity, MTX route of administration, FA dose or detailed comorbidity data, all of which may influence adherence and tolerability. Finally, the questionnaire assessed patients’ perceptions of same-day administration rather than actual adherence under such a regimen.

Despite these limitations, this large patient-centred survey identifies a potentially overlooked component of MTX management. The results do not demonstrate that simultaneous MTX and FA administration improves adherence, nor do they establish that this strategy is preferable to current practice. Rather, they identify self-reported FA omission in a meaningful proportion of patients and show that many patients consider regimen simplification worth evaluating. Folate supplementation has an established role in reducing MTX-related toxicity [11, 12], while preclinical data from our group suggest that concomitant MTX and FA administration does not impair MTX efficacy compared with separate administration [7]. Together, these findings provide a rationale for a prospective randomised trial comparing same-day with delayed FA administration. Such a study should assess both adherence and tolerability while also evaluating MTX effectiveness and accounting for FA dose, MTX route, treatment duration and disease activity.

## Supplementary Material

rkag109_Supplementary_Data

## Data Availability

The data underlying this article will be shared on reasonable request to the corresponding author.
