# Supplementary material for: Patient perspectives on simultaneous methotrexate–folic acid intake to improve adherence in rheumatoid arthritis: a cross-sectional survey
Source: Rheumatol Adv Pract. 2026 Oct 8;10(4):rkag109. doi: 10.1093/rap/rkag109 (PMC13654197; doi:10.1093/rap/rkag109)
Supplement: Supplementary file 1 [file rkag109_supplementary_data.docx]

**Supplementary Table S1.** Patterns of folic acid omission and post-methotrexate nausea among patients reporting omissions

|  |  | **MTX duration** | | | |  |
| --- | --- | --- | --- | --- | --- | --- |
|  | **Overall**  **N=247** | **0-2 years**  **N=19** | **2-10 years**  **N=89** | **>10 years**  **N=139** | **p-values** |  |
| **Frequency of FA omission** | | | | | 0.4 |  |
| All the time | 2 (0.8%) |  | 1 (1.1%) | 1 (0.7%) |  |  |
| Regularly | 23 (9.3%) | 2 (10.5%) | 9 (10.1%) | 12 (8.6%) |  |  |
| Sometimes | 40 (16.2%) | 4 (21.1%) | 18 (20.2%) | 18 (12.9%) |  |  |
| Occasionally | 87 (35.2%) | 3 (15.8%) | 29 (32.6%) | 55 (39.6%) |  |  |
| Never or rarely | 95 (38.5%) | 10 (52.6%) | 32 (36.0%) | 53 (38.1%) |  |  |
|  | | | | |  |  |
| **Nausea within 48 h after MTX** | | | | | 0.3 |  |
| Yes | 130 (52.6%) | 13 (68.4%) | 49 (55.1%) | 68 (48.9%) |  |  |
| No | 117 (47.4%) | 6 (31.6%) | 40 (44.9%) | 71 (51.1%) |  |  |
| **Increased nausea after FA omission** | | | | | 0.064 |  |
| Yes | 40 (16.2%) | 6 (31.6%) | 17 (19.1%) | 17 (12.2%) |  |  |
| No | 207 (83.8%) | 13 (68.4%) | 72 (80.9%) | 122 (87.8%) |  |  |
|  | | | | |  |  |

*N: number (percentages); MTX: methotrexate; FA: folic acid; p-values of Fisher’s exact test.*
